# Recombinant antibodies for specific detection of clostridial [Fe-Fe] hydrogenases

**DOI:** 10.1038/srep36034

**Published:** 2016-10-27

**Authors:** Rahul Mangayil, Matti Karp, Urpo Lamminmäki, Ville Santala

**Affiliations:** 1 Tampere University of Technology, Department of Chemistry and Bioengineering, Tampere, 33720, Finland; 2 University of Turku, Department of Biotechnology, Turku, 20014, Finland

## Abstract

Biological hydrogen production is based on activity of specific enzymes called hydrogenases. Hydrogenases are oxygen sensitive metalloenzymes containing Ni and/or Fe atoms at the active site, catalyzing reversible reduction of protons. Generally, [Fe-Fe] hydrogenases prefer proton reduction to molecular hydrogen, a potential energy carrier molecule that can be produced by bioprocesses in sustainable manner. Thus, monitoring tools have been developed to study the relationship between [Fe-Fe] hydrogenases and biohydrogen production in bioreactors at DNA and RNA levels. In the present study, novel molecular tools are introduced for quantitative monitoring of clostridial [Fe-Fe] hydrogenases at the protein level. Aerobic and anaerobic biopanning (for inactive and active [Fe-Fe] hydrogenase, respectively) of phage displayed single-chain variable fragment (scFv) antibody libraries aided in isolating nine potential scFvs. The enriched antibodies demonstrated high specificity towards *Clostridium* spp. [Fe-Fe] hydrogenases allowing detection from pure and mixed cultures. Additionally, the antibodies showed different binding characteristics towards hydrogenase catalytic states, providing a possible means for functional detection of clostridial [Fe-Fe] hydrogenases. From hydrogenase-antibody interaction studies we observed that though antibody binding reduced the enzyme catalytic activity, it facilitated to retain hydrogen evolution from oxygen exposed hydrogenases.

Depletion of traditional energy reserves, global warming and increased environmental pollution have strongly urged for alternative energy sources. Hydrogen (H_2_) is considered an alternative energy carrier due to its high energy yield, low heating value and non-polluting emission^1^. To increase sustainability in energy production, H_2_ can be produced biologically by dark fermentation using organic wastes as substrates in bioprocesses^2^.

Hydrogenases are the key enzyme involved in the metabolism of molecular H_2_. The enzyme is grouped into three classes based on the metal cofactor present at the active site, namely [Fe-Fe], [Ni-Fe] and [Fe] hydrogenases. In general, hydrogenases catalyze the reversible conversion of dihydrogen to protons and electrons through the reaction, *H*_*2*_ ↔ 2*H*^+^ + 2*e*^−^ 3. However, the oxygen (O_2_) sensitivity of hydrogenase is a limitation for some practical applications^4^. Studies indicate that O_2_ enters to the enzyme active site through the gas migration channels and on binding to the [Fe] moiety at the active site, generates reactive O_2_ species that destroys the enzyme catalytic function^5^,^6^,^7^.

Among the hydrogenase classes, [Fe-Fe] hydrogenases favors in proton reduction to molecular H_2_ and exhibits the highest catalytic activity^4^. This makes [Fe-Fe] hydrogenases the natural target for molecular prospections in biohydrogen production systems^8^. In bioprocess, quantitative monitoring of hydrogenase enzymes is important and can provide more in-sight on the process performance. Several groups have described tools for hydrogenase monitoring at DNA and RNA levels and reported how quantitative analyses of hydrogenases can reveal important clues about the bioprocess performance^8^,^9^,^10^. Previously our group reported the means to quantify *Clostridium butyricum* [Fe-Fe] hydrogenase at gene and transcript levels in an open bioprocess system^10^. Later on, the application of quantitative PCR and melting curve analysis of clostridial [Fe-Fe] hydrogenase as monitoring tools in an open bioreactor, assisting in elucidating biohydrogen production and changes in the functional community during the open fermentation process was investigated^8^. The relationship between H_2_ production kinetics and gene transcript levels of several *Clostridium* spp. isolated from continuous stirred tank reactor was investigated by Morra *et al.*^9^. The authors reported similar transcriptional profiles, but different H_2_ evolution kinetics among the isolates indicating a post-transcriptional down regulation of clostridial hydrogenases^9^. In the present study, to add another level for molecular prospections of hydrogenase enzyme in biological processes, we describe the isolation of specific antibodies for clostridial [Fe-Fe] hydrogenases.

Display of proteins or peptide libraries on the surface of filamentous phages, invented by Smith in 1985, has become a widely applied and powerful tool for screening antibodies for different molecular targets^11^. With the help of phage display, screening conditions can be adjusted according to specific needs. More specifically, phage displayed libraries have been used in protein engineering^12^, protein-protein interaction studies^13^, epitope mapping^14^, specific screening and quantification^15^, recognizing function-specific^16^ and diverse protein conformations^17^,^18^ of aerobic enzymes. However, screening of antibodies specific towards O_2_ labile targets has been rarely conducted. Bruggeman *et al.*^19^, have reported successful isolation of recombinant antibodies specific towards O_2_ sensitive flavin. Here, we report the enrichment of recombinant antibodies with specific binding characteristics towards [Fe-Fe] hydrogenases from *Clostridium* spp. using aerobic and anaerobic biopanning techniques.

## Results

### Biopanning for anti-hydrogenase antibodies

Biopanning of phage displayed antibody libraries were conducted to enrich and isolate scFvs specific towards catalytically active and inactive *Clostridium acetobutylicum* [Fe-Fe] hydrogenases. Streptavidin/avidin paramagnetic beads and streptavidin/neutravidin plates were chosen as the binding platforms for antibody panning of chemically biotinylated inactive and active hydrogenases, respectively. The biopanning surfaces were alternated at each panning round to avoid unspecific enrichment. Prior to biopanning, the purity of His-tag purified *C. acetobutylicum* hydrogenases were analyzed by SDS-PAGE (see Supplementary Fig. S1).

Under anoxic conditions, [Fe-Fe] hydrogenases are capable of reducing protons to molecular H_2_. However, upon exposure to O_2_, the catalytic activities of hydrogenase enzyme are irreversibly inactivated. Consequently, prior to the panning against active hydrogenases, the buffers were purged with nitrogen gas and stored in an anaerobic glove box. The antibody panning was conducted under strict anoxic conditions in anaerobic glove box. In the case for inactive hydrogenases, the biopanning was performed aerobically with O_2_ saturated buffers. The percentage ratio of output to input phages from biopanning panning rounds indicated clear phage enrichments (see Supplementary Table S1).

Cloning the enriched scFv genes from pEB32X phagemid vector into pAK600 expression vector allowed screening antibodies specific towards the target antigens. Ninety four (from inactive panning, see Supplementary Fig. S2) and ninety two (from active panning, see Supplementary Fig. S3) single clones were randomly selected and tested for antigen binding by alkaline phosphatase assay. Based on the signal level, twelve and eight scFvs that specifically recognized inactive and active hydrogenases, respectively, were selected. In the following text, the selected antibodies will be specified by the clone number and suffixes ‘In’ and ‘Ac’, representing antibodies screened from inactive and active biopanning, respectively. Sequencing revealed that all the 8 antibodies recognizing active hydrogenase (7Ac, 23Ac, 31Ac, 43Ac, 49Ac, 59Ac, 82Ac and 88Ac) were different and among the twelve inactive hydrogenase specific antibodies only two unique clones (7In and 48In) were found. The amino acid sequences of complementary determining regions in the selected scFvs are presented in Supplementary Table S2.

### Antibodies recognize hydrogenases from Clostridium spp. and differentiate between enzyme functional forms

The scFv antibody clones were investigated for their binding towards hydrogenases in *Clostridium* spp. by sandwich immunoassay. The supernatant obtained after centrifuging the lysed cells (lysate) were employed as immunoassay antigens. The scFv genes (48In, 7Ac, 23Ac, 31Ac, 43Ac, 49Ac, 59Ac, 82Ac and 88Ac) (see Supplementary Table S3) and were cloned to pAK400cb vector, which allows to express the scFvs as an N-terminal fusion with biotin carboxyl carrier protein (BCCP) domain in the cytoplasm of *Escherichia coli* Origami B^20^. A biotin moiety is enzymatically added to BCCP by the cell, and hereby the scFv-BCCP fusion affords a straightforward way for the production of biotinylated antibodies for efficient immobilization. With the exception of 7In, expression and *in vivo* biotinylation of scFv-BCCP fusion proteins was successful in the redox modified *E. coli* strain.

**Figure 1:**
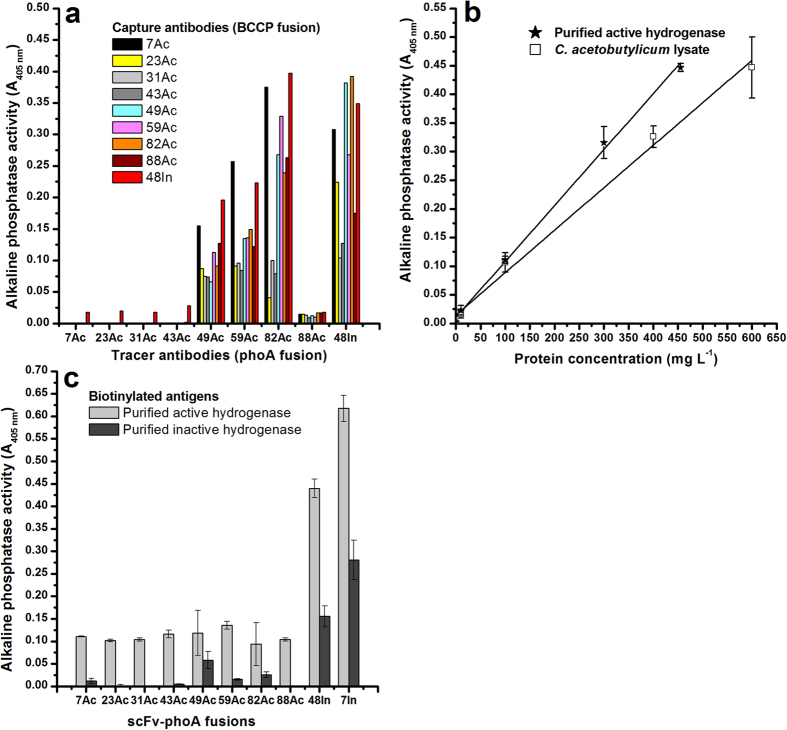
Sandwich and one-step immunoassays of *C. acetobutylicum* hydrogenases (affinity purified and in crude cell lysates) with the isolated scFvs. The above graphs depicts (**a**) the binding specificities of the enriched antibody clones (7Ac, 23Ac, 31Ac, 43Ac, 49Ac, 59Ac, 82Ac, 88Ac and 48In) towards *C. acetobutylicum* lysate, (**b**) dose response immunoassay with the 7Ac-BCCP and 82Ac-phoA immunoassay pairs against *C. acetobutylicum* lysate and purified *C. acetobutylicum* hydrogenase. The data points from *C. acetobutylicum* lysate (Empty square), and purified active hydrogenase (filled star) were fitted linearly (The solid line represents the linear regression; R-square values: 0.99, purified active hydrogenase; 0.99, *C. acetobutylicum* lysate) and (**c**) one-step immunoassay demonstrating the binding specificities of the enriched scFvs’ towards affinity purified and biotinylated active and inactive hydrogenases. The data represents mean absorbance values (A_405 nm_) subtracted from the background signals (Sandwich immunoassay, *E. coli* XL1 and Origami B lysates containing empty pAK600 and pAK400cb plasmids; One-step Immunoassay, *E. coli* XL1 lysate containing empty pAK600 plasmid). The error bars indicate standard deviation of averaged data (A_405_) from triplicate microtiter wells.

Upon confirmation of hydrogenase activity (see Supplementary Table S4), sandwich immunoassay was performed with *C. acetobutylicum* lysate, scFv-BCCP (capture antibody) and scFv-phoA (tracer antibody) fusion proteins. The lysates of *E. coli* XL1 and Origami B carrying empty plasmids were included as negative controls. Clearly detectable absorbance (A_405_ nm) values were observed for all scFv-BCCP fusions with 49Ac-phoA, 59Ac-phoA and 82Ac-phoA fusions as tracer antibodies. Whereas, negligible or very low signals were observed with 7Ac, 23Ac, 31Ac, 43Ac and 88Ac as tracer antibodies (Fig. 1a). High absorbance values were observed when 48In was used either as capture or tracer antibody. Based on the results, 82Ac and 48In were the best performing tracer antibodies. The order of capture scFv-BCCP fusions in accordance to the signal obtained with 82Ac as tracer was: 48In > 7Ac > 59Ac > 49Ac > 88Ac > 82Ac > 31Ac > 43Ac > 23Ac.

The assay configuration based on the antibody pair 7Ac (capture) and 82Ac (tracer) was tested against varying concentrations of *C. acetobutylicum* lysate and recombinant active *C. acetobutylicum* [Fe-Fe] hydrogenase (Fig. 1b). *E. coli* XL1 and Origami B lysates containing pAK600 and pAK400cb vectors devoid of scFv genes were included as negative controls. A linear fit was observed with escalated concentrations of purified active hydrogenase (R-square, 0.99) and *C. acetobutylicum* lysate (R-square, 0.99) concentrations (Fig. 1b).

To confirm the binding specificity of scFvs towards functionally different forms of [Fe-Fe] hydrogenases, the scFv-phoA fusions (7Ac, 23Ac, 31Ac, 43Ac, 49Ac, 59Ac, 82Ac, 88Ac, 7In and 48In) was tested in one-step immunoassay for their specificity towards the biotinylated recombinant active and inactive *C. acetobutylicum* hydrogenases (see Supplementary Table S4). The antibodies exhibited different binding characteristics towards catalytically active and inactive hydrogenases. From the experimental data presented in Fig. 1c, it can be inferred that antibodies 7Ac, 23Ac, 31Ac, 43Ac, 59Ac, 82Ac and 88Ac exclusively recognized the active hydrogenase, whereas this was not the case for the clones 49Ac, 7In and 48In.

From bioprocess monitoring perspective, it is important to test whether the selected antibodies are capable of recognizing hydrogenases from closely related *Clostridium* spp. For that reason, *C. butyricum*, an efficient mesophilic H_2_ producer, was chosen in this study. The amino acid sequence similarity of [Fe-Fe] hydrogenases from *C. acetobutylicum* (AAB03723) and *C. butyricum* (ACD62594.1) by ClustalW2 was identified to be 67% (see Supplementary Fig. S4).

**Figure 2:**
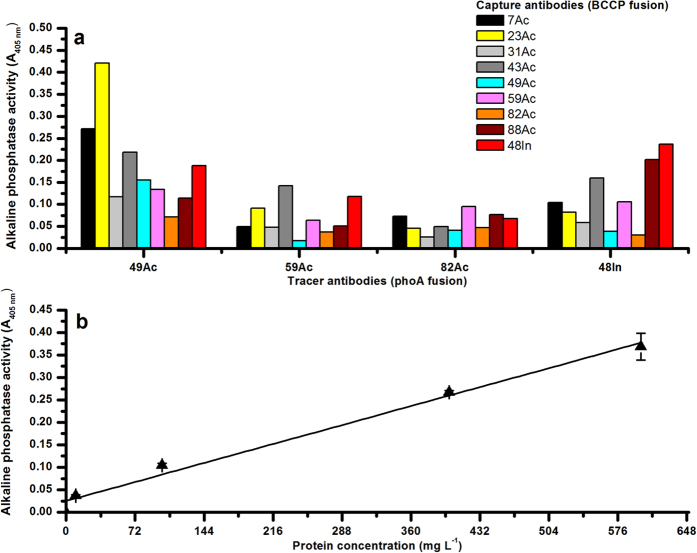
Sandwich immunoassay with scFv-BCCP and scFv-phoA fusion proteins as capture and tracer antibodies, respectively. The above graphs depict (**a**) the binding specificities of the enriched anti-hydrogenase antibodies towards *C. butyricum* lysate and (**b**) linear regression (R^2^ = 0.98) data from sandwich immunoassay using 23Ac-BCCP and 49Ac-phoA immunoassay pairs and *C. butyricum* lysate. The plotted data represents blank (*E. coli* lysates containing empty pAK400cb and pAK600 plasmids) subtracted mean absorbance values (A_405 nm_) and standard deviations from triplicate microtiter wells.

After confirming hydrogenase activity in the lysate (see Supplementary Table S4), the sandwich immunoassay configurations recognizing the *C. acetobutylicum* [Fe-Fe] hydrogenase were investigated for their capacity to detect [Fe-Fe] hydrogenase from *C. butyricum*. The assay was performed in an anaerobic glove box with the inclusion of blank *E. coli* XL1-pAK600 and Origami B-pAK400cb carrying empty plasmids as negative controls. Interestingly, absorbance signals were obtained from all the binder combinations tested (Fig. 2a). Among the tracer antibodies tested, 49Ac-phoA gave the highest signal with most of the capture antibodies tested and the immunoassay pair exhibiting the highest signal was 23Ac-BCCP and 49Ac-phoA. The order of capture antibodies (BCCP fusions) in accordance to the signal obtained with 49Ac tracer is: 23Ac > 7Ac > 43Ac > 48In > 31Ac > 49Ac > 59Ac > 88Ac > 82Ac.

Dose-dependent immunoassay with 23Ac-BCCP (capture antibody) and 49Ac-phoA (tracer antibody) was performed on streptavidin coated microtiter wells against increasing concentrations of *C. butyricum* lysate. Figure 2b depicts the mean absorbance signal obtained from triplicate microtiter well readings with 0–600 mg L^−1^ of antigen. A linear fit with an R-square value of 0.98 in absorbance response observed during the immunoassay.

### scFvs bind selectively to [Fe-Fe] hydrogenases

In order to obtain purified antibodies for the following experiments, the scFv genes were cloned from pAK600 into pLK06H vector and transformed into *E. coli* XL1 strain. The cells were induced with 100 μM IPTG overnight and lysed. The filtered lysates were purified (His-tag based immobilized metal affinity chromatography) and buffer exchanged with 1X phosphate buffered saline (PBS). The purified proteins were analyzed by SDS-PAGE (see Supplementary Fig. S5).

**Figure 3:**
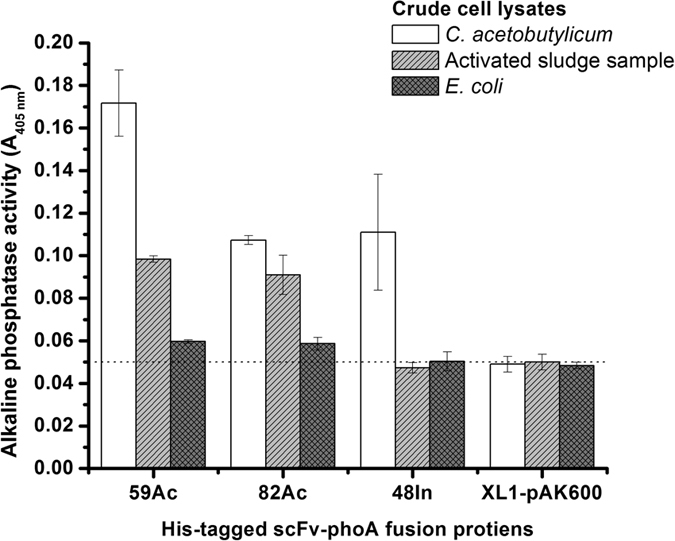
One-step immunoassay with affinity purified scFv-phoA fusion proteins [59Ac, 82Ac, 48In and XL1-pAK600 (empty vector)] and biotinylated antigens (*C. acetobutylicum*, activated sludge and *E. coli* lysates). The horizontal dashed line indicates alkaline phosphatase activity level from immunoassay tests with the empty vector and respective antigen lysates. The graphical data represents mean absorbance values (A_405 nm_) and error bars from triplicate microtiter wells.

As the antibodies employed in study showed different expression profiles, scFv-phoA-(6X)His fusions discerned as strong bands in SDS-PAGE (59Ac, 82Ac and 48In) were selected as tracer antibodies for one-step immunoassay with chemically biotinylated antigen lysates [*C. acetobutylicum* (positive control), enriched activated sludge sample^21^ and *E. coli* XL1 (negative control)]. Background phoA activity was checked by including His-tag purified empty *E. coli* XL1-pAK600 as tracer antibody. The mean alkaline phosphatase signals from triplicate well readings corresponding to antigen-antibody interaction are presented in Fig. 3. Similar to the previous results, the scFvs recognized hydrogenases in the positive control (*C. acetobutylicum* lysate). Relative to data obtained from XL1-pAK600 lysate, purified 59Ac, 82Ac and 48In antibodies generated 2–2.5 fold higher phoA activities with *C. acetobutylicum* lysate as assay antigen. Inclusion of an enriched activated sludge sample (predominated with *Clostridium* spp.)^21^, enabled to investigate whether the antibodies recognized [Fe-Fe] hydrogenases from mixed microbial consortia. Antibodies 59Ac and 82Ac recognized the antigen in activated sludge sample and on comparison with XL1-pAK600 yielded higher phoA activity. Alkaline phosphatase activity by phoA gene was not recorded for any anti-hydrogenase antibodies with *E. coli* lysate as immunoassay antigen. These results indicate that the antibodies are capable of recognizing hydrogenases from mixed microbial cultures. Additionally, the experimental data confirms the antibody binding specificity towards [Fe-Fe] hydrogenases.

### Antigen-antibody interactions impart changes in enzyme characteristics

The effect of scFv binding on hydrogenase enzyme characteristics was investigated. The changes in enzyme activities imparted by antigen-antibody interactions were studied using recombinant hydrogenases and scFv immobilized on streptavidin beads (7Ac, 59Ac, 82Ac and 48In as fused to BCCP domain). Prior to experiment, the presence of scFv on streptavidin beads was confirmed by sandwich immunoassay with purified hydrogenase antigen and 82Ac-phoA as tracer antibody (see Supplementary Table S5). Lysates from cells harboring empty vectors (Origami-pAK400cb and XL1-pAK600) were used as assay controls. After confirmation, the streptavidin beads coated with scFvs were incubated anaerobically with catalytically active recombinant hydrogenase in rotating mixer [1 hour at room temperature (RT)] in two sets. Until the wash step, both the sets were prepared in similar conditions. The washes were conducted in parallel for both the sets, i.e. in Set 1, antigen-scFv-beads were washed with anaerobic TBT-0.05 amended with 20 mM sodium dithionite and for Set 2 using aerobic TBT-0.05 buffer. As washing removes the unbound hydrogenases, H_2_ evolution is performed solely by the scFv bound hydrogenases.

In the experiment, two controls were included. Bead control (purified hydrogenases coated to streptavidin beads, i.e. no scFvs’) demonstrates the background activity of antigen coated to antibody-null streptavidin beads. Enzyme control (purified hydrogenases in aerobic and anaerobic 50 mM Tris-HCl) enabled to identify the original enzyme catalytic activity and validate hydrogenase inactivation on exposure to O_2_ saturated buffer. The mean H_2_ productions (nmol) and standard deviations from triplicate methyl viologen oxidation experiments (in 2 ml reaction buffer, anoxic 50 mM Tris-HCl supplemented with 5 mM methyl viologen as electron donor) are presented in Table 1. In Bead control, Set 1 produced negligible enzyme activity and headspace H_2_ content was absent when washed with aerobic TBT-0.05 (Bead control, Set 2). Similar O_2_ mediated enzyme inactivation was observed in Enzyme control (Set 2). In comparison with Enzyme control, the hydrogenase catalytic activity was greatly affected with scFv binding (see Supplementary Table S6). The interactions with scFvs resulted in 3–5 fold reduced H_2_ production. Interestingly, upon exposure to O_2_ the antigen-scFv complex (Set 2) retained methyl viologen oxidation property, producing comparable H_2_ to the corresponding sample in Set 1. The percentage drop in enzyme activity upon O_2_ exposure for antigen bound 7Ac, 59Ac, 82Ac and 48In antibodies were 25%, 11%, 54% and 73%, respectively. The t-Test results for the experimental data (n = 3; Table 1) postulates that the drop in enzyme activity observed for hydrogenase bound 82Ac and 48In antibodies is statistically significant (82Ac, p = 0.027; 48In, p = 0.025). Whereas, antigen bound to 7Ac and 59Ac antibodies imparted statistically insignificant (7Ac, p = 0.189; 59Ac, p = 0.649) decrease in hydrogenase catalytic activity.

## Discussion

H_2_ gas has a pivotal role in many biological processes as energy carrier. Since hydrogenases are the key enzymes involved in H_2_ metabolism in biological systems, they are rational targets for such molecular level explorations^8^,^9^,^10^. The present study aims in employing anti-hydrogenase antibodies as tools for protein level monitoring of hydrogenases.

Enrichment and isolation of antibodies recognizing O_2_ labile proteins is a challenging task. The target enzyme in this study is irreversibly inactivated in presence of O_2_, and such process might be reflected as changes in the epitopes potentially recognized the antibodies. Inactivation of [Fe-Fe] hydrogenase on exposure to O_2_ undergoes several steps. Under aerobic conditions O_2_ enters into the protein structure and binds to enzyme catalytic site through gas migration channels, generating reactive oxygen species^4^,^5^,^6^,^7^. This imparts irreversible oxidative damage of electron transfer domains and active site. Thus, aerobic and anaerobic biopanning of phage displayed antibody libraries were conducted against purified inactive and active *C. acetobutylicum* hydrogenases, respectively. From the results, we observed increased phage enrichment from aerobic biopanning (see Supplementary Table S1) and amino acid sequence similarities among antibodies enriched against inactive hydrogenases: only two unique clones were found. This may indicate the loss of antigenic epitopes as the hydrogenase structural integrity is affected during the inactivation process^22^.

In order to employ the enriched scFvs’ as monitoring tool in bioprocess systems, we studied their binding characteristics towards hydrogenases from different *Clostridium* spp. and mixed microbial communities^23^. Firstly, the scFvs enriched against *C. acetobutylicum* [Fe-Fe] hydrogenases were capable of recognizing the target antigen from *C. butyricum*. This binding characteristics apparently reflects the relatively high degree of similarity among clostridial [Fe-Fe] hydrogenases at protein level^24^. The amino acid sequence involved in the formation of the active site at the C-terminal region of [Fe-Fe] hydrogenases is highly conserved among clostridial type [Fe-Fe] hydrogenases^25^,^26^. By selecting optimal antibody pairs, more specific hydrogenase recognition can be obtained: antibody pair (7Ac-BCCP and 82Ac-phoA) showed stronger binding characteristics towards *C. acetobutylicum* [Fe-Fe] hydrogenase compared with [Fe-Fe] hydrogenase from *C. butyricum* (Figs 1a and 2a). Secondly, the immunoassay results (Fig. 3) demonstrates that the scFvs clear recognition profile [Fe-Fe] hydrogenases. These interpretations points towards the capacity of scFvs from this study as monitoring tools in hydrogen-fermenting systems.

Previous reports indicate that scFvs interactions with active enzymes may demonstrate reduced or inhibitory effects on the enzyme catalytic property^27^,^28^. Similar antibody mediated changes in enzyme properties were observed in this study. On comparison with Enzyme control (see Supplementary Table S6), hydrogenases bound to antibody showed reduced catalytic activities. When exposed to O_2_, hydrogenases bound to 82Ac and 48In antibodies conferred a drop in H_2_ production. Very interesting finding was that the hydrogenase bound by 7Ac and 59Ac scFv retained methyl viologen oxidation property in the studied conditions (Table 1). This inflection in enzyme characteristics allows to speculate that the antibodies 7Ac and 59Ac recognized epitopes near to hydrogenase gas migration channels. However, this finding requires more experimental validations and will be scrutinized in future investigations.

This is the first study to report successful generation of recombinant antibodies recognizing anaerobic microbial [Fe-Fe] hydrogenases. The antibodies described here can be useful for the protein level monitoring purposes when applied together with other molecular monitoring tools. Future investigations on epitope mapping, elucidating scFv binding kinetics and antibody interactions with [Fe-Fe] hydrogenases from a phylogenetically distinct source will illuminate immense potentials in biotechnological applications, for example in affinity-purification of [Fe-Fe] hydrogenases for structural studies and attempts to improve the O_2_ tolerance of [Fe-Fe] hydrogenases by specific molecular interactions.

## Materials and Methods

The methods for recombinant expression and purification of *C. acetobutylicum* hydrogenase, preparation of bacterial cell lysates and methyl viologen oxidation assay are provided in Supplementary Information.

### Phage libraries, helper phage and solid surfaces

High-diversity phage-displayed antibody libraries and VCS-M13 helper phage (Stratagene, USA) were used in the biopanning process^29^. Dynabeads MyOne Streptavidin T1 (Invitrogen, USA) and avidin paramagnetic beads (Gentaur Molecular Products, Belgium) were selected as solid surfaces for aerobic panning (inactive hydrogenase). For anaerobic panning (active hydrogenase) streptavidin (Kaivogen, Finland) and neutravidin (Pierce NeutrAvidin, Thermo Scientific, USA) coated microtiter plates were used.

### Bacterial strains, plasmids and culture media

*E. coli* XL1 (Stratagene, USA) strain was used for phage infection, production of enriched phages and expression of scFv-phoA fusion protein in pAK600 vector. The pAK600 vector assists in periplasmic expression of scFv-bacterial alkaline phosphatase (phoA) fusion protein. Origami B (Novagen, USA) strain harboring pAK400cb vector was used for expressing scFv-BCCP fusion proteins for sandwich immunoassay^20^. Recombinant antibody expression in *E. coli* XL1 was conducted by cloning scFv genes into pLK06H vector using *Sfi*I restriction sites. pLK06H is an ampicillin-resistant version of pAK600 vector constructed to introduce hexhistag at the C-terminal end of phoA gene^29^.

Antibody recognition towards hydrogenases in bacterial lysates was conducted using *C. acetobutylicum* DSM 792, *C. butyricum* DSM 2478, activated sludge predominated with Clostridium spp.^21^ and *E. coli* XL1 lysates. *E. coli* BL21 (DE3) ∆*iscR* pFEGA was used for recombinant expression of *C. acetobutylicum* hydrogenase^30^. Low salt Lysogeny agar (LA) plates and Super broth (SB) medium were prepared as described^31^. The biopanning reagents such as Polyethylene Glycol 8000-Sodium Chloride [PEG_8000_-NaCl], Tris Base-Sodium Chloride [TBS; pH 7.5], TBS-Bovine Serum Albumin-Tween 20 (0.5%) [TBT-0.5; pH 7.5], TBT-0.05 (pH 7.5) and TBS-Sodium azide (0.02% w/v)-Tween 20 (0.05%) [TSAT-0.05; pH 7.5] solutions were prepared as described previously^32^.

### Biopanning

His-tag purified hydrogenases and *E. coli* BL21 (DE3) lysate were chemically biotinylated (EZ-Link Sulfo-NHS-Biotin, Thermo Scientific, USA) and buffer exchanged twice by NAP^TM^ column (Sephadex^TM^-G-25 DNA grade, GE healthcare, UK) with 1X PBS. Biotinylation and buffer exchange of purified active hydrogenases was performed in an anaerobic glove box (Don Whitley Scientific, UK) and stored anaerobically at 4 °C (Anaerocult, Merk, Germany).

Mixed libraries (phage diversity, 5 × 10^12^ c.f.u.) were used for the first round of phage display. Streptavidin beads and streptavidin wells were chosen as biopanning platforms for inactive and active hydrogenases, respectively. Subtractive panning was performed by incubating the phage stock with selection platforms and biotinylated blank BL21 (DE3) lysate (70 mg L^−1^). For the blank lysate, *E. coli* BL21 (DE3) was grown and lysed by lysozyme (1 g L^−1^) and freeze-thaw treatments in the presence of 1X protease inhibitor cocktail mix (cOmplete EDTA-free, Roche, Germany). The protein concentrations were measured by Quickstart^TM^ Bradford assay kit (Bio-rad, USA). In anaerobic panning, the input phage library was prepared by incubating the phage stock individually with empty streptavidin wells and BL21 (DE3) lysate for 15 minutes followed with phage precipitation using PEG_8000_-NaCl solution. For aerobic panning, the subtractive panning was performed by incubating phage stock with BL21 (DE3) lysate coated on streptavidin beads for 5 minutes and thereafter collected. The following biopanning steps were performed as schematically presented in Supplementary Fig. S6.

In the second round, neutravidin coated microtiter plate wells and avidin coated beads were used as selection platforms with reduced phage library (5 × 10^11^ c.f.u.) and protein concentrations (10 mg L^−1^). For the final round, streptavidin coated microtiter wells and beads were used with more stringent panning conditions (5 × 10^10^ c.f.u and 5 mg L^−1^).

### Identification of anti-hydrogenase antibodies

The enriched scFv genes (from 3^rd^ biopanning round) were sub-cloned ‘en-masse’ into pAK600 vector using *Sfi*I restriction sites and transformed to *E. coli* XL1 cells. Individual clones were cultured in SB medium and scFv expression was induced with 250 μM IPTG (OD_600_ nm 0.6–0.7) and grown for 16–20 hours (23 °C and 300 rpm). The cells were lysed as previously described and lysate containing scFv-phoA fusion protein was used in the following assays.

First in the screening, chemically biotinylated recombinant *C. acetobutylicum* hydrogenases (70 mg L^−1^) were added into pre-washed (TBT-0.05) streptavidin wells and incubated under aerobic (for inactive hydrogenase) or anaerobic (for active hydrogenase) conditions at RT for 1 hour (intermittent shaking). The wells were washed thrice with TBT-0.05 and *E. coli* XL1 lysates containing scFv-phoA fusion proteins were incubated with immobilized hydrogenases. To determine any unspecific binding, scFv-phoA fusions were incubated separately with empty streptavidin wells and biotinylated BL21 (DE3) lysate. *E. coli* XL1-pAK600 lysates lacking antibody gene was also included as control to determine background phoA activity. Following the incubation, the wells were washed as described previously and alkaline phosphatase substrate (p-nitrophenyl phosphate, Sigma) was added. The plates were incubated at 37 °C with slow shaking for 1–2 hours. The scFv-phoA fusions with binding towards hydrogenase (measured as alkaline phosphatase activity at A_405_ nm) were selected and sequenced (Forward primer M13R-pUC(−40), Macrogen).

Screening for antibodies specific towards active hydrogenase was performed in an anaerobic glove box. The buffers for anaerobic immunoassay screening were pre-sparged with N_2_ for 2 hours and stored in the anaerobic chamber.

### Antibody purification

The scFv genes cloned from pAK600 into pLK06H vector were transformed to *E. coli* XL1 cells. Single clones cultured in SB medium were induced with 100 μM IPTG (16–20 hours at 23 °C/300 rpm). His-tag purification of scFv-phoA fusion proteins was conducted as described by the manufacturer (Novagen, USA).

For antigen-antibody interaction studies, scFv-BCCP fusion proteins (7Ac, 59Ac, 82Ac and 48In) were purified using streptavidin beads. One milliliter of *E.coli* lysate containing scFv-BCCP fusions were mixed with 20 μl of pre-washed beads (PBS) at RT for 3 hours in a rotating mixer. The beads were washed four times with 1 ml TBT-0.05 buffer and stored in 200 μl TSAT-0.05 buffer at 4 °C. Presence of antibody on streptavidin beads were confirmed by sandwich immunoassay with purified hydrogenase, scFv-BCCP coated beads and 82Ac-phoA as antigen, capture and tracer antibodies respectively. Empty Origami B-pAK400cb and XL1-pAK600 strains were included as immunoassay negative controls.

### Immunoassay

In order to obtain biotinylated capture antibodies for sandwich immunoassay, scFv genes [one from aerobic panning (48In) and eight from anaerobic panning (7Ac, 23Ac, 31Ac, 43Ac, 49Ac, 59Ac, 82Ac and 88Ac)] were amplified using rm13_1 and rm13_2 primers (see Supplementary Table S3). The amplified product was sub-cloned into pAK400cb vector using *Nde*I and *EcoR*I restriction sites and transformed into Origami B.

*E. coli* XL1-scFv-phoA (tracer antibody expression) and Origami B-scFv-BCCP (capture antibody expression) strains were grown in SB medium supplemented with 0.1% glucose, 10 mg L^−1^ tetracycline, 25 mg L^−1^ chloramphenicol and 15 mg L^−1^ kanamycin (Origami B). Overexpression of scFv-BCCP and scFv-phoA fusion proteins were initiated at OD_600_ nm 0.6–0.7 with 250 μM IPTG (23 °C/300 rpm) for 24 and 48 hours, respectively. Following cell lysis, the immunoassay experiments were conducted in anaerobic glove box as described previously. The capture antibodies (scFv-BCCP fusions), antigens (*C. acetobutylicum* and *C. butyricum* lysates) and tracer antibodies (scFv-phoA fusions) were added to streptavidin wells in the order. Empty *E. coli* XL1-pAK600 and Origami B-pAK400cb were included as negative control.

One-step immunoassay was conducted to observe antibody specificities towards [Fe-Fe] hydrogenases in environmental samples. Briefly, the immunoassay was conducted using streptavidin well individually immobilized with 70 mg L^−1^ biotinylated *C. acetobutylicum* (positive control), enriched activated sludge sample and *E. coli* XL1 lysates (negative control) and 10 mg L^−1^ His-tag purified scFv-phoA fusions (59Ac, 82Ac and 48In). His-tag purified *E. coli* XL1 lysate was included as the negative control. The alkaline phosphatase activities were calculated as mean signals (A_405nm_) obtained from triplicate well readings.

The dose dependent immunoassay was conducted under anaerobic conditions using the antibody pairs that exhibited maximal signal for respective target antigens (7Ac-BCCP:82Ac-phoA, *C. acetobutylicum* lysate and purified active hydrogenase; 23Ac-BCCP:49Ac-phoA, *C. butyricum* lysate). Empty *E. coli* XL1-pAK600 and Origami B-pAK400cb lysates were included as negative controls.

### Antigen-antibody interactions on enzyme characteristics

Antibodies expressing scFv-BCCP fusions (7Ac, 59Ac, 82Ac and 48In) were grown, induced, lysed and coated to 20 μl streptavidin beads in two Sets. The beads were transferred to anaerobic glove box. Each scFv bound beads were incubated with 40 ng of purified hydrogenase (1 hour/RT). The unbound hydrogenases were removed by washing the beads (4X) in parallel - Set 1 with TBT-0.05 containing 20 mM sodium dithionite and Set 2 with aerobic TBT-0.05. Care was taken to prevent O_2_ escape from wash buffer by maintaining O_2_-saturated buffer outside the glove box, keeping the container lid tightly sealed and checking for O_2_ presence with anoxic water containing 0.002 gL^−1^ resazurin. After the wash steps, the beads were resuspended in anoxic 1X PBS and the hydrogenase activities were analyzed by methyl viologen oxidation assay. The assay included two controls: Bead control (streptavidin beads coated with purified hydrogenases) and Enzyme control [recombinant hydrogenase (40 ng) prepared in aerobic and anaerobic 50 mM Tris-HCl (pH 8.0)].

### Statistical analysis

The data from antigen-antibody interaction studies (individual sample in Set 1 to the corresponding sample in Set 2) were statistically analyzed by Two-Sample t-Test Assuming Unequal Variances using Data Analysis function in Microsoft Excel 2007. The p value [P(T <= t) two-tail]  < 0.05 indicates statistical significance.

## Additional Information

**How to cite this article**: Mangayil, R. *et al.* Recombinant antibodies for specific detection of clostridial [Fe-Fe] hydrogenases. *Sci. Rep.* **6**, 36034; doi: 10.1038/srep36034 (2016).

**Publisher’s note:** Springer Nature remains neutral with regard to jurisdictional claims in published maps and institutional affiliations.

## Supplementary Material

Supplementary Information

## Figures and Tables

**Table 1:** Perturbations on hydrogenase enzyme characteristics by the antibody interactions.

Immobilized scFv	H_2_ production (nmol)^a^		P(T <= t) two-tail^b^
	Set 1 (After anaerobic wash step)	Set 2 (After aerobic wash step)	
7Ac	188 ± 43	141 ± 19	0.181
59Ac	168 ± 52	150 ± 37	0.649
82Ac	255 ± 24	116 ± 55	0.027
48In	239 ± 25	66 ± 42	0.025
Bead control	14 ± 7	0	ND
Enzyme control	1041 ± 145	0	0.006

^a^H_2_ evolution activity of streptavidin-scFv-antigen complexes exposed to aerobic and anaerobic buffers by methyl viologen oxidation assay. The H_2_ production and standard deviations presented are mean data from 3 independent assays.

^b^The Two-Sample t-Test Assuming Unequal Variances (p value) was performed using the H_2_ production (nmol) data from bead immobilized scFv-hydrogenase complex in Set 1 (anaerobic wash) to the corresponding sample in Set 2 (aerobic wash).

ND, Not determined.
