# Supplementary material for: Recombinant antibodies for specific detection of clostridial [Fe-Fe] hydrogenases
Source: Sci Rep. 2016 Oct 27;6:36034. doi: 10.1038/srep36034 (PMC5081567; doi:10.1038/srep36034)
Supplement: Supplementary Information [file srep36034-s1.pdf]

SUPPLEMENTARY INFORMATION

Recombinant antibodies for specific detection of clostridial [Fe-Fe] hydrogenases

Rahul Mangayil [1], Matti Karp [1], Urpo Lamminmäki [2], Ville Santala [1]

[1] Department of Chemistry and Bioengineering, Tampere University of Technology, Tampere, Finland

[2] Department of Biotechnology, University of Turku, Turku, Finland

Corresponding author – Rahul Mangayil (email: rahul.mangayil@tut.fi)

Supplementary Figures

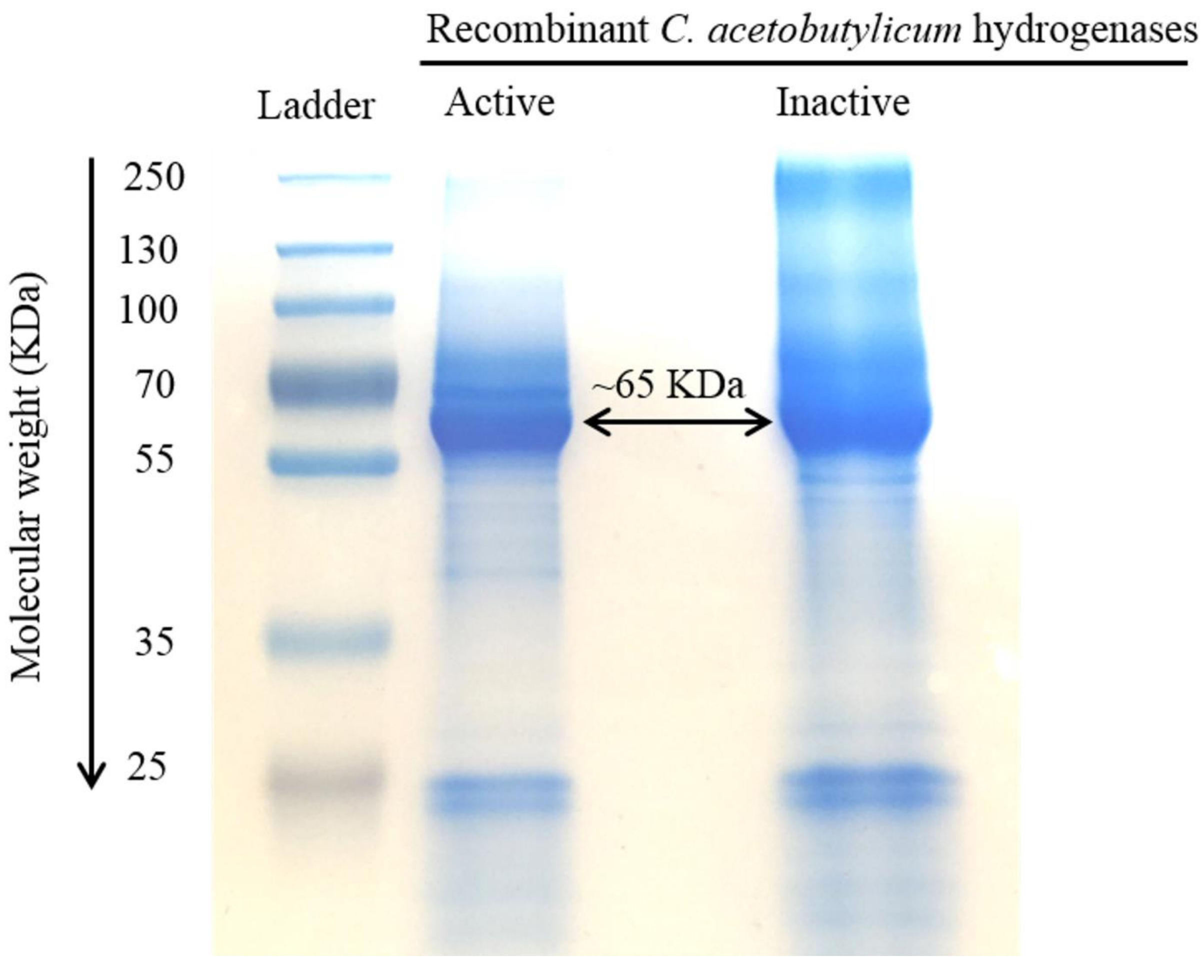

Supplementary Figure S1. SDS-PAGE (12%) analysis of catalytically active and inactive His-tag purified *C. acetobutylicum* hydrogenases.

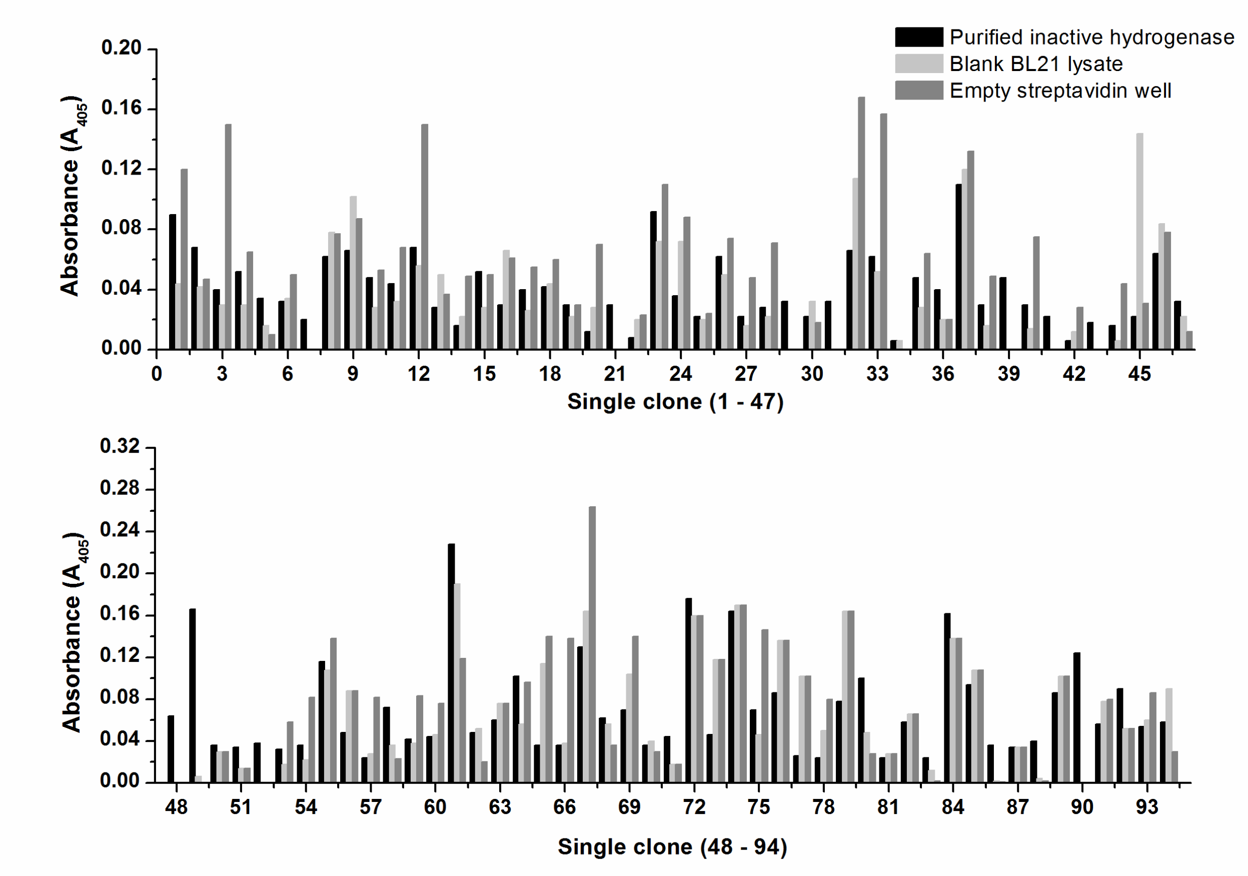

Supplementary Figure S2. One-site immunoassay for initial screening of antibodies recognizing inactive hydrogenase. Antibody genes from 3rd panning round were sub-cloned to pAK600 expression vector. Ninety four random clones were induced with 250µM IPTG to express as scFv-phoA antibody fusions. The antibody fusions were tested for their recognition profile towards target antigen, selection platform (streptavidin coated microtitre wells) and blank BL21 (DE3) lysate.

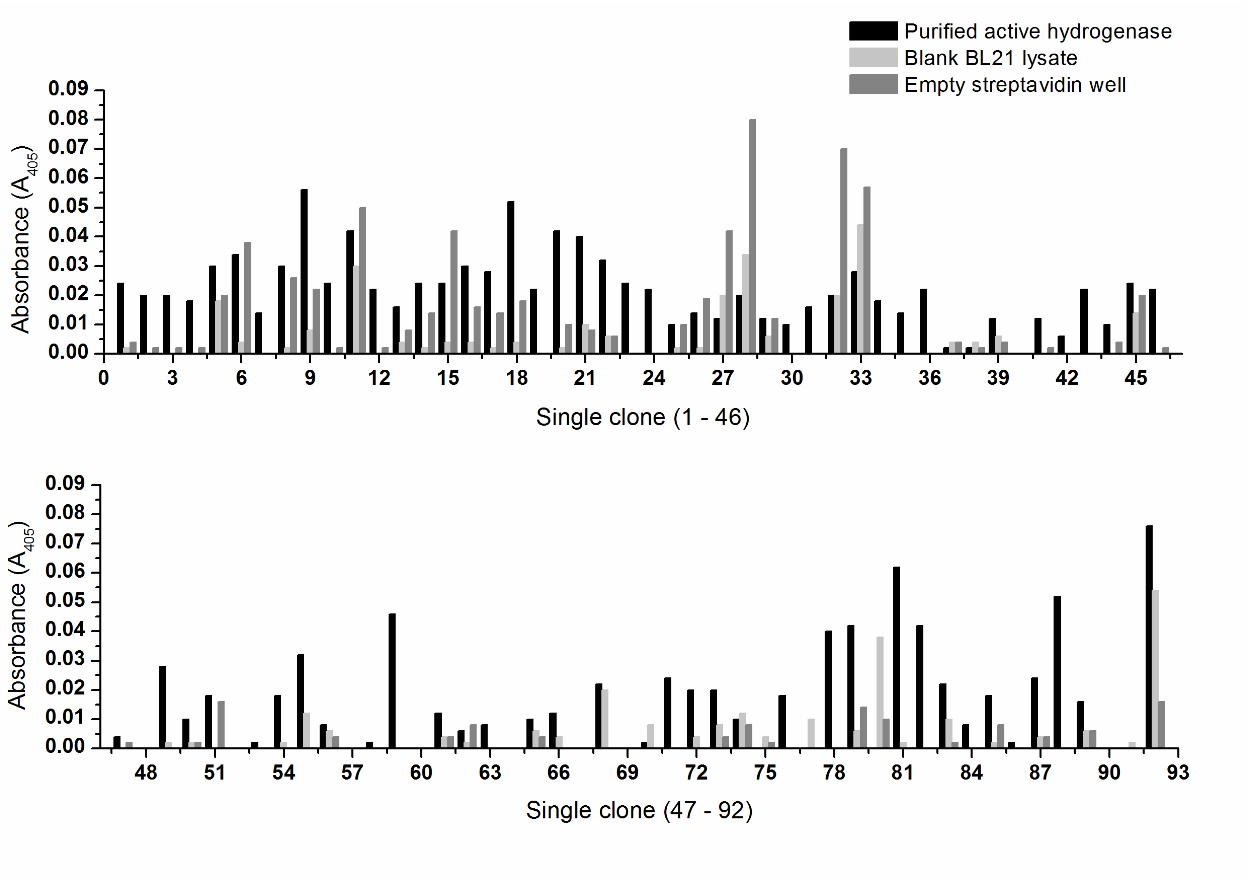

Supplementary Figure S3. One-site alkaline phosphatase assay to screen for antibodies recognizing active hydrogenase. Antibody genes from 3rd panning round were sub-cloned to pAK600 expression vector. Ninety two random clones were induced with 250µM IPTG to express as scFv-phoA antibody fusions. The antibody fusions were tested for their recognition profile towards target antigen, selection platform (streptavidin coated microtitre wells) and blank BL21 (DE3) lysate.

```
1    mktiilngnevhtdkditilelarennvdiptlcflkdcgnfgkcgvcmvevegkg-fra   59  Acetobutylicum
1    minliidekn vqvpegtsiiqaaldngidipslcylkecsnvgkcgvcavevegkkvlsl   60  Butyricum
     * .:*:: ::*:. :  :*:: * :* :***:**:**:*.*.****** ******   :

60   acvakvedgmvintesdevkerikkrvsmlldkhefkcgqcsrrenceflklviktkaka  119  Acetobutylicum
61   acltkveegmvvrtnteatqervkmrvaalldkhefkcgpcprretceflklviktkaka  120  Butyricum
     **::***:***:.*::: .:**:* **: ********** * ***.**************

120  skpflpedkdalvdnrskaividrskcvlcgrcvaackqhtstcsiqfikkdgqravgtv  179  Acetobutylicum
121  nkpflptdktefllddrsksitidrskcvlcgrcvaackektgtgnieisgkgpdrkvqai  180  Butyricum
     .***** **  ::*:***:*.*****************::*.* .*::  *  :* * ::

180  ddvclddstcllcgqcviacpvaalkekshiekvqealndpkkhvivamapsvrtamgel  239  Acetobutylicum
181  egkcfdetncllcgqcvaacpvaalnekphidrvkealedpnkhvivamapavrtsmgel  240  Butyricum
     :  *:*::.******** *******:** **::*:***:**:*********:***:****

240  fkmgygkdvtgklytalrmlgfdkvfdinfgadmtimeeatellgrvknngpfpmftscc  299  Acetobutylicum
241  fkmgygvdvtgklysslrqlgfdkvfdinfgadmtimeeatefierinnngpfpmftscc  300  Butyricum
     ****** *******::** ***********************:: *::************

300  pawvrlaqnyhpelldnlssakspqqifgtasktyypsisgiapedvytvtimpcndkky  359  Acetobutylicum
301  pawvrqaenyypellgnlssakspqqifgaasktyyptvegldpksvytvtimpctakky  360  Butyricum
     ***** *:**:**** *************:*******::.*: *:.*********. ***

360  eadipfmetnslrdidasltt relakmikdakikfadledgevdpamgtysgagaifgat  419  Acetobutylicum
361  eadrtemeneglrnidavlttrelakmikdakinfatledekadpamgeytgagvifgat  420  Butyricum
     ***   **.:.**:*** ***************:** *** :.***** *:***.*****

420  ggvmeaairsakdfaenkelenvdytevrgfkgikeaeveiagnklnvavingasnffef  479  Acetobutylicum
421  ggvmeaalrtakdfvedkdlidieykqvrgldgikeatveiggknynvavingsanltkf  480  Butyricum
     *******:*:****.*:*:* :::*.:***:.***** ***.*:: *******::*: :*

480  mksgkmnekqyhfievmacpggcingggqphvnaldrenvdyrklrasvlynqdknvlsk  539  Acetobutylicum
481  veggqmdekqyhfvevmscpggcvngggqphvaakdrdtanvravrasvlynqdktav-k  539  Butyricum
     ::.*:*:******:***:*****:******** * **:..: * :**********..: *

540  rkshdnpaiikmydsyfgkpgeglahkllhvkytkdknvskhe  582  Acetobutylicum
540  rkshknealmkmyneymgmpgqhkahellhlkytk--------  574  Butyricum
     ****.* *::***:.*:* **:  **:***:****
```

Supplementary Figure S4. Amino acid sequence alignment of [Fe-Fe] hydrogenases from *C. acetobutylicum* (GenBank accession number: AAB03723.1) and *C. butyricum* (GenBank accession number: ACD62594.1).

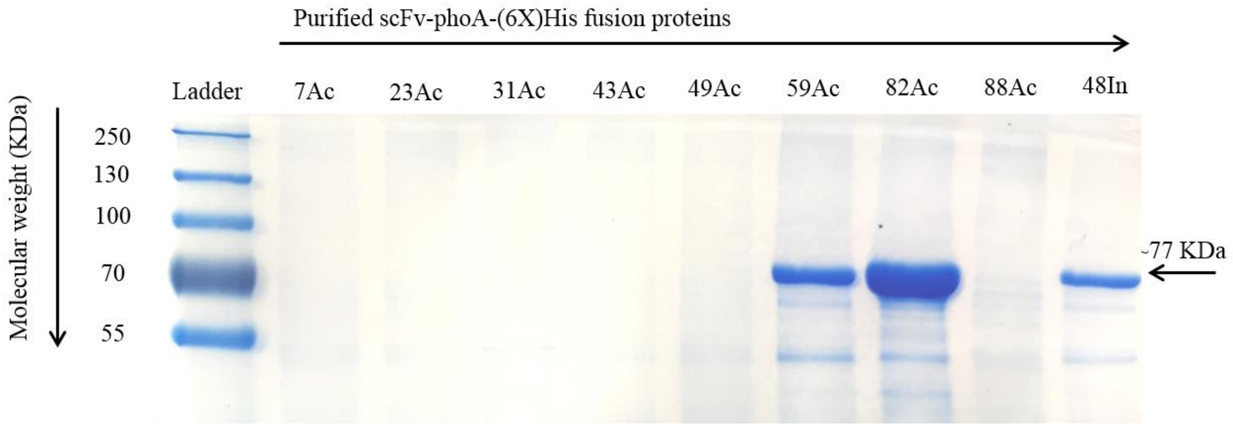

Supplementary Figure S5. IPTG induced expression profile of scFv-phoA-(6X)His fusion proteins by 12% SDS-PAGE.

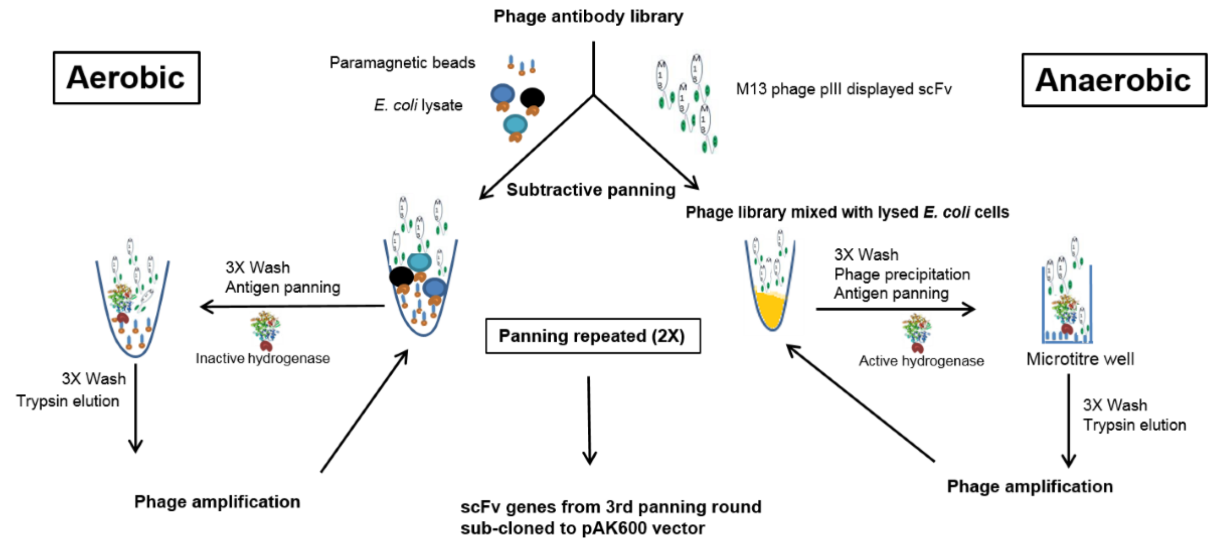

Supplementary Figure S6. Flowchart displaying aerobic and anaerobic phage display steps, cloning, and immunoassay studies in the order performed, aiming in selection of Clostridial [Fe-Fe] hydrogenase specific antibodies. The biopanning steps were performed with mixed libraries and inactive and active hydrogenase as target antigens. Subtractive planning round was performed by incubating the phage stock with the selection platforms and biotinylated blank BL21 (DE3) lysate. Phages unspecific to background proteins and selection platform were then incubated with biotinylated target antigens (70 mg L^{-1}) for 1 hour at RT. Unspecific phages were removed by washing the panning platforms twice with TBT-0.5 and once with TSAT-0.05. Bound phages were eluted with 50 mg L-1 trypsin in TSAT-0.05 at 37˚C for 30 minutes. New phage stock for subsequent panning rounds were prepared by infecting fresh XL1-Blue cells (OD_{600} 0.5 – 0.6) with the eluate and VCS M13 helper phage. The number of eluted phages from the panning round was estimated by plating the infected *E. coli* XL1-Blue cells in LA-agar amended with tetracycline (10 mg L^{-1}) and chloramphenicol (25 mg L^{-1}).

Supplementary Tables

Supplementary Table S1. Phage enrichment from biopanning rounds with purified inactive and active [Fe-Fe] hydrogenases.

**Panning rounds**	**Input (c.f.u./ml)**	**Output (c.f.u./ml)**	**Output/Input (%)**	**Fold increase to 1st round**
**Purified inactive *C. acetobutylicum* hydrogenase**				
1	5×10^{12}	9×10^{6}	0.000018	
2	5×10^{11}	6.8×10^{6}	0.000272	15.11
3	5×10^{10}	2.47×10^{7}	0.00394	218.89
**Purified active *C. acetobutylicum* hydrogenase**				
1	5×10^{12}	6.07×10^{7}	0.00121	
2	5×10^{11}	6.8×10^{6}	0.0065	5.35
3	5×10^{10}	2.47×10^{7}	0.056	46.11

Supplementary Table S2. Single clone sequence alignment of complementary determining regions (CDR)

**Antibody clones**[a]	**Light chain**		**Heavy chain**		
	CDR-L1	CDR-L3	CDR-H1	CDR-H2	CDR-H3
**Clone selected against inactive hydrogenase**					
48In	YLN	LQDYYTPF	SYSMD	GITPSGGSTY	ASYKDWGFDY
**Clones selected against active hydrogenase**					
7Ac	PLN	LQNTSDPF	SYLMS	RISPSGGSTD	ARGQYNGPDY
23Ac	NLA	QQSYSTPW	SDVMH	SINPNTGYTT	ASWTRDSLDI
31Ac	YLN	QQAYYIPH	SYLMQ	WIASSGGSTD	ARHDDPAD
43Ac	PLG	LQGYYIPY	SYSMD	EINPSGGSTN	ARWYDWDGVFDY
49Ac	NLA	QQSSSTPW	DYWMH	EIRPSTGSTN	ARWDFDY
59Ac	NLA	QQSSSLPW	NYGMH	EIWPSSGYTY	ARSWNTGEGWLDY
82Ac	NLA	QQSYSTPW	NYGMH	EINTVSGETY	ARKSDQFDY
88Ac	SLN	LQWNSSPY	SYLMH	SIAPSGGSTD	ARGGDFDI

[a] CDR domain alignment of antibodies with identical amino acid sequences enriched against inactive hydrogenase and 7In binder is excluded in the table.

Supplementary Table S3. Primer sequences for the amplification of scFv genes from pAK600 plasmid

**Primer**	**Sequence**
rm13_1	5’- CAATCATATGGAAATTGTGCTGACCCAATCTC -3’
rm13_2	5’-TCCGGAATTCGGCCCCCGAGGCC-3’

Supplementary Table S4. Measured hydrogenase activities in this study

**Sample**	**Total protein concentration (mg/ml)**	**Specific activity**[a] (µmol H_2 min^{-1} mg^{-1})
Hydrogenase activity measured before and after His-tag purification of IPTG induced E. coli BL21 (DE3) ΔiscR-pFEGA lysate		
E. coli lysate (Before purification)	2.8	51 ± 15
Active *C. acetobutylicum* hydrogenase (from anaerobic His-tag purification)	0.8	904 ± 38
Inactive *C. acetobutylicum* hydrogenase (from aerobic His-tag purification)	0.7	0
Hydrogenase activities of anaerobically cultivated pure cell lysates		
C. acetobutylicum	1.4	368 ± 138
C. butyricum	1.2	435 ± 12
Enriched activated sludge population	1.4	562 ± 134
E. coli XL1 Blue	1.1	61 ± 35

[a] Specific activities experiments were conducted, in triplicates, with 30 ng of hydrogenase (as purified or lysates) in 50 mM Tris-Hcl supplemented with 20 mM sodium dithionite and 5 mM methyl viologen as electron donor.

Supplementary Table S5. Validating the presence of scFv-BCCP fusions on streptavidin beads by sandwich immunoassay [a]

**Capture antibody (scFv-BCCP)**	**Alkaline phosphatase activity ($A_{405\ nm}$)** [b]
7Ac	0.13 ± 0.01
59Ac	0.10 ± 0.03
82Ac	0.13 ± 0.06
48In	0.11 ± 0.01
Empty XL1-pAK400cb	0.04 ± 0.00

[a] For the immunoassay, His-tag purified *C. acetobutylicum* hydrogenase and 82Ac-phoA were antigen and capture antibody, respectively.

[b] The mean absorbance values and standard deviations were calculated from the signals obtained from triplicate well readings.

Supplementary Table S6. Effect of antibody binding on hydrogenase catalytic activity.

**Purified scFv-phoA-(6X)His fusions** [a]	**Specific activity (μmol H_2 min^{-1} mg^{-1})** [c]	**P(T<=t) two-tail from Two-Sample t-Test Assuming Unequal Variances (p value)** [d]
59Ac	274.1±71.79	0.0003
82Ac	586.3±92.1	0.0365
48In	633.4±148.9	0.1962
E.coli XL1-pAK600 [b]	735.1±25.7	0.6308
Enzyme control [b]	779.8±22.1	

a Under anaerobic conditions, purified hydrogenase and antibodies were added to anoxic 50 mM Tris-Hcl supplemented with 5 mM methyl viologen. The vials were incubated at RT for 1 hour in rocking platform.

[b] Positive and negative controls included in this study are Enzyme control (His-tag purified hydrogenase alone, 30 ng) and hydrogenase incubated with XL1-pAK600 lysate (14 μg/ml), respectively.

[c] The error bar indicates standard deviations from three independent experiments.

[d] The student-t test was performed using the specific activity data from antigen-antibody complex in solution with that from the Enzyme control.

Supplementary Methods

Recombinant expression and purification of *C. acetobutylicum* hydrogenase

For recombinant expression of clostridial hydrogenase, *E. coli* BL21 (DE3) Δ*iscR*-pFEGA strain was grown aerobically in LB Miller medium supplemented with 0.4% glucose and 2 mM ferric citrate (37˚C and 200 rpm) until an optical density (OD_{600}) of 0.6 – 0.7. One millimolar cysteine hydrochloride and 500 µM IPTG (Fermentas, Lithuania) were added upon transfer to anaerobic glove box. The culture bottles were capped, sealed and purged with nitrogen (N_2) for 10 minutes. The cultures were then incubated anaerobically (26˚C and 200 rpm) for 16 – 20 hours. The cells were harvested by centrifugation (23,708×*g*, 5 minutes, 4˚C) and washed twice with 1X His-tag purification binding buffer prepared per manufacturer directions (Novagen, USA). Cell lysis was performed under anaerobic conditions by lysozyme (1 g L^{-1}) and three freeze-thaw treatments. The cell debris was removed by centrifugation (23,708×*g*, 25 minutes, 4˚C).

The His-tag purification buffer stocks were prepared as mentioned by the manufacturer (Novagen, USA). Purification of inactive hydrogenase was performed by applying the filtered supernatant (0.2 µm membrane) to 1 ml nickel charged His-bind column with O_2 saturated buffers. Following the lysate addition, the resin was washed with 10 column volume (CV) of 1X wash buffer and the recombinant protein was eluted with 2.5 CV of 1X elution buffer. The elution fraction containing inactive hydrogenase was buffer exchanged by NAP^{TM} column with 1X PBS and stored at 4°C.

Purification of catalytically active recombinant [Fe-Fe] hydrogenase was executed in an anaerobic glove box. The His-tag purification buffers and 1X PBS were purged with N_2 for 2

hours, capped, sealed and stored in anaerobic glove box. Prior to experiments, the hydrogenase activity of the purified protein was analyzed by methyl viologen oxidation assay. The purification of [Fe-Fe] hydrogenases was conducted similarly as mentioned for purifying inactive recombinant hydrogenases under strict anaerobic conditions. The purified enzyme was buffer exchanged with anoxic 1X PBS, sealed and stored anaerobically at 4°C (Anaerocult, Merk, Germany).

The protein purities and concentration were analyzed with SDS-PAGE and Quickstart™ Bradford assay kit (Bio-rad, USA), respectively.

Growth and preparation of bacterial crude lysates

C. acetobutylicum DSM 792, *C. butyricum* DSM 2478, *E. coli* XL1 and bacterial community in activated sludge were grown anaerobically (37˚C and 150 rpm) in 250 ml Reinforced Clostridial Medium (Merk, Germany) for 16-20 hours. The cells were harvested (23,708×*g*, 5 minutes, 4˚C), washed with anoxic 1X PBS and re-suspended in the same buffer. Cell lysis was performed as described previously. The lysed cells were removed by centrifugation (23,708×*g*, 25 minutes, 4˚C) and the supernatant was used as crude lysate. The lysates were then analyzed for hydrogenase activity by methyl viologen oxidation assay and stored anaerobically at 4˚C. For one-step immunoassay, the bacterial lysates were chemically biotinylated and buffer exchanged twice with anoxic 1X PBS to remove unreacted biotin.

Methyl viologen oxidation assay

Hydrogenase samples, either purified or crude cell lysate (30 - 35 ng), were added to 50 mM Tris-HCl (pH 8.0) supplemented with 5 mM methyl viologen and 20 mM sodium dithionite

under anoxic environment. To measure background H_2, a glove box blank was prepared with equal liquid volume to that of the sample vials. Hydrogenase activity was initiated by incubating the vials at 37˚C for 1 hour. The H_2 amounts in the headspace were analyzed using a Gas chromatograph (GC-2014, Shimadzu GC) and N_2 (Instrument N_2 5.0) was used as carrier gas. The GC was equipped with PORAPAK column (2 m*2 mm) and thermal conductivity detector. The carrier gas flow rate and operating temperatures of the column, detector and oven were 20 ml/min, 80 °C, 110 °C and 80 °C, respectively. The H_2 content from GC was converted to millimoles (mmol) using gas-law constant. The enzyme specific activities were calculated by subtracting the glove box H_2 content from the experimental data and dividing the produced H_2 (mmol) with the incubation time and protein amount.
